# Radial EBUS versus CT-guided needle biopsy for evaluation of solitary pulmonary nodules

**DOI:** 10.18632/oncotarget.23952

**Published:** 2018-01-04

**Authors:** Wei Wang, Like Yu, Yuchao Wang, Qian Zhang, Chuanzhen Chi, Ping Zhan, Chunhua Xu

**Affiliations:** ^1^ Endoscopic Center of Nanjing Chest Hospital, Nanjing, 210009, Jiangsu, China; ^2^ Clinical Center of Nanjing Respiratory Diseases and Imaging, Nanjing, 210009, China; ^3^ Department of Respiratory Medicine, The Affiliated Hospital of Southeast University, Nanjing, Jiangsu 210009, China

**Keywords:** solitary pulmonary nodule, endobroncheal ultrasonography guided bronchoscopy, ct guided percutaneous needle biopsy, diagnostic yield, complication

## Abstract

**Objective:**

This study is aimed to compare the diagnostic yield, complications and influencing factors between Radial endobroncheal ultrasonography guided bronchoscopy(R-EBUS) and CT-guided needle biopsy (CT-PNB), for evaluation of solitary pulmonary nodules(SPNs).

**Matrials and Methods:**

160 cases of consecutive patients with SPNs were enrolled and divided into R-EBUS and CT-PNB groups randomly. The diagnostic yield, complications and influencing factors between the two groups were evaluated.

**Results:**

Sensitivity of R-EBUS for malignancy was 73.7% (42/57) and for benign, was 43.5% (10/23), overall diagnostic accuracy was 65% (52/80). In CT-PNB group, overall diagnostic accuracy was 85% (68/80), sensitivity for malignancy was 87.9% (51/58), and for benign was 81.0% (17/21), respectively. Both overall diagnostic yield and incidence of complications in CT-PNB group were higher than those in R-EBUS group (*P* = 0.006, *P* = 0.002). In R-EBUS group, the factors affecting diagnostic yield were size (*P* = 0.027), the distance between SPNs and pleura (*P* = 0.031) and the location of the probe to lesions (*P* = 0.009). In CT-PNB group, the distance from the lesions to pleura was correlated with the incidence of pneumothorax (*P* = 0.001) and pulmonary haemorrhage (*P* = 0.042). The location of SPNs were adjacent to great vessels was another influencing factor for pulmonary haemorrhage (*P* = 0.042).

**Conclusions:**

Both R-EBUS and CT-PNB are valuable tools for diagnosis. SPNs located in medial 1/2 of lung field, or were adjacent to great vessels may be fit for R-EBUS. Those SPNs located in lateral 1/2 of lung field, near to pleura or with less vessels around may be more suitable for CT-PNB.

## INTRODUCTION

Single pulmonary nodules (SPNs) is defined as single, isolated lesion of circular or ovoid shape, with the diameter of ≤ 30 mm, which is located within the lung parenchyma, surrounded entirely by gas-containing lung tissue and not accompanied wiht hilar enlargement, pleural effusion or lung atelectasis [1]. With the wide application of high-resolution spiral CT [2, 3], SPNs are detected at an increasing rate [4–6]. Quite a number of them represented malignant and were forthcoming lung cancer in early stage, thus the pathological diagnosis of SPN is urgently needed to comfirm as early as possible [7]. Although computed tomography guided percutaneous needle biopsy (CT-PNB) has been regarded as an useful tool for SPNs pathological diagonsis with the highest diagnostic accuracy reported as more than 90% [8], complications such as pneumothorax and pulmonary haemorrhage are relatively high too [9, 10]. Conventional flexible bronchoscopy is a safe technique means for diagnosing pulmonary lesion, however, its application is restricted because its limited scope. Many SPN lesions are located outside bronchial lumen thus could not be invisible. In recent years, the development of radial endobronchial ultrasound (R-EBUS) technology have overcome this shortcoming. It can be maximized for SPNs that are away from the pleura. Using R-EBUS, vast majority of peripheral pulmonary nodules can be detected [11]. However, the comparison between R-EBUS and CT-PNB have been relatively rare. Recently some studies compared cost between R-EBUS and CT-PNB by economic analysis [12, 13]. To our knowledge, few researchers have been reported about the diagnostic yield of R-EBUS compared with CT-PNB in evaluation of peripheral pulmonary lesion(PPL) [10, 14]. Alough the influencing factors in the diagnostic yield of R-EUBS were studied [15, 16], the influencing factors for complications of the two means have not well established. In order to provide more resonable choices in evaluating SPNs, we tried in this study to perform a randomized trial to compare the diagnostic values, complications and influencing factors to them between R-EBUS and CT-PNB the two methods.

## RESULTS

### Patients demogrpahics and SPNs characteristics of R-EBUS and CT-PNB groups

Table 1 listed the data as age and gender of patients, location and lobar position of SPNs between R-EBUS and CT-PNB groups, No significant statistical difference in these baseline characteristics was detected between the two groups (*P* > 0.05). The average diameter of SPN (mean ± SD) was (2.17 ± 0.31) cm in the R-EBUS group and (2.09 ± 0.30) cm, in the CT-PNB group. (added to Table 1, blod red section). No statistical difference between the mean diameters of SPNs in the two groups Statistical results also showed that there is no difference in the constituent ratio of diameter size between the two groups.

**Table 1:** Patients demogrpahics and SPNs characteristics of R-EBUS and CT-PNB groups

	R-EBUS group (*n*)	CT-PNB group (*n*)	*P*-value
Patients number	80	80	
Age (mean ± SD) (year)	58.67 ± 13.55	59.03 ± 13.06	0.730
Gender (F/M)	48/32	45/35	0.749
Diameter of SPNs (mean ± SD) (cm)	2.17 ± 0.31	2.09 ± 0.30	0.441
Location of SPNs			
Medial 1/2 of lung field	19	23	0.472
Lateral 1/2 of lung field	61	57	
Lobar position			
RUL	19	17	> 0.05
RML	10	8	
RLL	12	14	
LUL	23	27	
LLL	16	14	

RUL, right upper lobe; LUS, left upper segment; RML, right middle lobe; RLL, right lower lobe; LLL, left lower lobe. et al.

### Pathological diagnosis of SPNs in R-EBUS group and CT-PNB group

As shown in the Table 2, Sensitivity of R-EBUS for malignancy was 73.7% (42/57) including adenocarcinoma, Squamous cell carcinoma, Small cell carcinoma, Metastatic carcinoma, pulmonaryand adenocarcinoma occupied the most proportion (38/42) of malignant SPNs. The sensitivity of R-EBUS for benign lesions was 43.5% (10/23) including tuberculosis, pneumonia, organic pneumonia, pulmonary fungal infection. The overall diagnostic accuracy in R-EBUS was 65% (52/80). In CT-PNB group, over diagnostic accuracy was 85% (68/80), sensitivity for malignancy was 87.9% (51/58), and for benign was 81.0% (17/21), respectively.

**Table 2:** Pathological findings in patients and Subsequent methods to establish final diagnosis

Procedure	Pathologic findings		*n*	Methods for diagnosis established
**Diagnostic**				
R-EBUS (*n =* 52)	Malignant	Adenocarcinoma	38	
		Squamous cell carcinoma	2	
		Small cell carcinoma	1	
		Metastatic carcinoma	1	
	benign	Tuberculosis	5	
		Pneumonia	2	
		Organic pneumonia	1	
		Pulmonary fungal infection	2	
CT-PNB (*n =* 68)	malignant	Adenocarcinoma	45	
		Squamous cell carcinoma	3	
		Small cell carcinoma	2	
		Metastatic carcinoma	1	
	benign	Tuberculosis	9	
		Pneumonia	2	
		Organic pneumonia	2	
		Pulmonary fungal infection	3	
		Pulmonary abscess	1	
Non-diagostic				
R-EBUS (*n =* 28)	malignant	Squamous cell carcinoma	2	VATS
		Large cell carcinoma	1	VATS
		Adenocarcinoma	11	VATS
		Metastatic carcinoma	1	VATS
	benign	Tuberculosis	7	VATS and antituberculous therapy
		Organic pneumonia	2	VATS
		Mycotic infection	3	antifungal therapy
		Hamartoma	1	VATS
CT-PNB (*n =* 12)	malignant	Adenocarcinoma	5	VATS
		Squamous cell carcinoma	1	VATS
		Metastatic carcinoma	1	VATS
		Tuberculosis	3	VATS and antituberculous therapy
	benign	Inflammation associated with pulmonary cyst	1	antibiotics
		Organic pneumonia	1	VATS

### Diagnostic yield and influencing factors between the R-EBUS and CT-PNB groups

Three factors affecting diagnostic yield in the R-EBUS group were listed as follows (Table 3): (a) size of SPNs. (b) the distance from the SPN lesion to pleura. (c) the relationship between the inserted probe and the SPN position.

**Table 3:** Comparison of diagnostic rates, incidence of complications and influencing factors between two groups (*n*[%])

Variables	R-EBUS group			CT-PNB group		
	Diagnostic yield	Pneumothorax	Hemorrhage	Diagnostic yield	Pneumothorax	Hemorrhage
Size of lesion						
> 8mm and ≤ 10 mm	0/1 (0%)	0/1 (0%)	0/1 (0%)	1/1 (100%)	1/1 (100%)	0/1 (0%)
> 10mm and ≤ 20 mm	16/32 (50.0%)	1/32 (3.1%)	2/32 (6.3%)	30/36 (83.3%)	8/36 (22.2%)	3/36 (8.3%)
> 20mm and ≤ 30 mm	36/47 (76.6%)	0/47 (0%)	2/47 (4.3%)	37/43 (86.1%)	5/43 (11.6%)	3/43 (6.4%)
*χ*^2^	4.862	-	0.016	0.000	0.922	0.040
*P-*value	0.027^*^	-	0.900	0.984	0.336	0.842
**Distance between** **SPN and pleura**						
≤ 20 mm	9/20 (45.0%)	0/20 (0%)	1/20 (5%)	21/24 (87.5%)	0/24 (0%)	0/24 (0%)
> 20 mm and ≤ 40 mm	26/38 (68.4%)	0/38 (0%)	1/38 (2.6%)	30/37 (81.1%)	5/37 (13.5%)	2/37 (5.4%)
> 40 mm	17/22 (77.3%)	1/22 (4.5%)	2/22 (9.1%)	17/21 (81.1%)	9/21 (42.9%)	4/21 (19.0%)
*χ*^2^	4.929	2.670	1.224	0.501	15.133	6.356
*P-*value	0.031^*^	0.263	0.542	0.778	0.001^*^	0.042α
**Relationship with** **great vessels**						
near	17/26 (65.4%)	0/26 (0%)	3/26 (11.5%)	20/23 (87.0%)	5/23 (21.7%)	5/23 (21.7%)
not near	35/54 (64.8%)	1/54 (1.9%)	1/54 (1.9%)	48/57 (84.2%)	9/57 (16.7%)	1/57 (1.8%)
*χ*^2^	0.040	-	1.727	0.001	0.095	6.773
*P-*value	0.841	-	0.189	0.972	0.757	0.009^*^
**Location of the probe**						
within	39/51 (76.5%)	0/51 (0%)	1/51 (2.0%)	-		
invisible or adjacent to	13/29 (44.8%)	1/29 (3.4%)	3/29 (10.3%)	-		
*χ*^2^	6.805	-	1.017	-		
*P-*value	0.009^*^	-	0.313	-		

^*^P < 0.01 ^α^*P* < 0.05

Further analysis was performed to indicating that in 3 subgroups with different SPNs size, those SPNs with the diameter between > 8 mm and ≤ 10 mm, were not diagnosed. Those SPN with diameter > 20 mm and ≤ 30 mm had higher diagnostic yield than that those with their diameter > 10 mm and ≤ 20 mm cases (*P* = 0.027).

Based on difference in distances from the lesion to pleura, we divided all patients with SPNs into three subgroups (≤ 20 mm, from 20 mm to 40 mm and > 40 mm). From the data in Table 3 we noticed that the longer the distance between the SPN lesion and pleura was, the higher the diagnostic yield were obtained with a statistical difference (*P* = 0.031).

**Figure 1:**
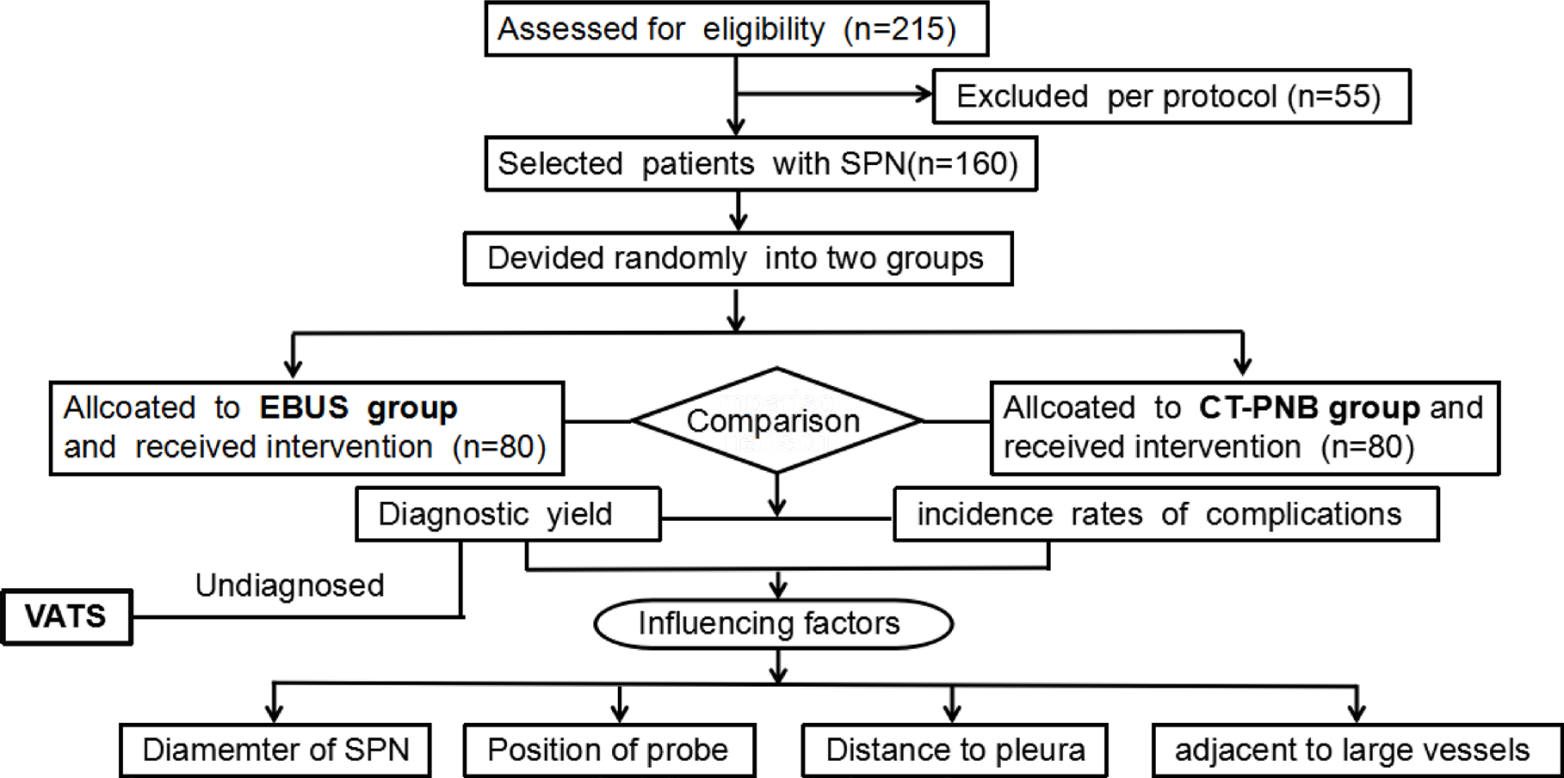
Flow diagram of the eligible patients and the interventional process of the study

According to the relationship between the inserted probe and the SPNs’ location, the ultrasonic images were categorized into three patterns: (a) invisible: abnormal echogenicity was not completely detected; (b) within: entire circumference of abnormal echogenicity was detected; (c) adjacent to: partial circumference of abnormal echogenicity was detected; (the picture A, B, C in Figure 1). Statistical analysis elaborated those cases with the probe exactly within the SPN lesion were more likely to be obtained positive diagnosis than those cases’ probes were adjacent or invisible to SPN lesions (*P* = 0.009).

Further logistic analysis results in Table 2 conveyed that the size of nodule and the distance from SPN to pleura were two influencing factors for the diagnostic yield in R-EBUS group (*P* = 0.027, *P* = 0.031). However, these two factors were not observed to influence the diagnostic accuracy in CT-PNB group (*P* = 0.984, *P* = 0.778).

Comparison of incidence of compliactions (pneumothorax and pulmonary hemorrhage) and influencing factors between the R-EBUS and CT-PNB groups.

As listed in Table 3, in CT-PNB group, the incidence of pneumothorax was 17.5% (14/80), 14 patients encountered pneumothorax including 1 patient requiring insertion of thoracotomy tube. In R-EBUS group, only 1 patient had pneumothorax with compressed lung volume < 5% and was relieved in 2 days through oxygen therapy, the rate of pneumothorax was 1.25% (1/80). The incidence of pneumothorax between these two groups was significant different (*P* = 0.001).

Another common complication is hemorrhage. In this study, 4 patients in R-EBUS group and 6 patients in CT-PNB group encountered pulmonary hemorrhage with no statistical difference in its incidence between two groups, (*P =* 0.900, and *P* = 0.842, respectively) (Table 3).

Further analysis showed in CT-PNB, no significant difference of incidence of pneumothorax among three subgroups according to size of SPNs. Base on the different distance from the SPN lesion to pleura, we found no pneumothorax occured in the subgroup in which the distance from SPN to pleura was ≤ 20 mm (0%, 0/24). However, a significant difference was found between the rest two subgroups in which distance from lesion to pleura ranged was 20 mm to 40 mm or > 40 mm (*P* = 0.001). Furthermore, the distance between the lesion and pleura was also another influencing factor for pulmonary hemorrhage (*P* = 0.042). The Table 3 also indecated that those SPNs’ location was near to great vessels would be more likely to cause pulmonary hemorrhage (*P* = 0.009).

## DISCUSSION

This trial was performed under randomization principle and baseline assessment. The baseline characteristics bwteen two groups of recruited patients showed no significant difference (all *P* > 0.05) (Table 1).

From Table 1 we can see in either R-EBUS or CT-PNB group, the Lobar positions of SPNs were similar which was consistent to previous documents [17, 18]. The rate of benign lesions in R-EBUS group was 28.75% (23/80) and in CT-PNB group was 26.25% (21/80), lower than those of malignant in the two groups (see in Table 2). We noticed that in our study, the benign lesions’ rate was higher compared some literature [10, 19], however, in Shinagawa N’s [20] study, all recruited patients with peripheral pulmonary lesions (PPLs) that were subsequently diagnosed as benign diseases. The difference of there studies indicated either malignant or benign lesions can present as SPNs. Because our study was a randomized pragmatic trial, so benign lesions occupied a certain proportion.

Our data demonstrated that the diagnostic yield in CT-PNB group were higher than that in R-EUBS group. The reason is that the guidence of CT scanning could make clear whether puncture needle had entered into the SPN lesion before biopsy, while R-EBUS’s guidence can hardly perform such real-time supervision. Therefore, the diagnostic yield with R-EBUS’s guidence was lower than with CT-PNB but still maintained the diagnostic accuracy of 65% [14].

As mentioned in our results, size of SPN was important factors affecting the diagnostic yield in R-EBUS group, which has been described by many other reports [14–16, 21]. When the size of SPNs was larger, the ultrasound probe of R-EBUS could be more easier to detect the lesion [22]. We found it interesting that no similar change took place in CT-PNB group, which were somewhat different from past studies [8]. We speculated that for SPNs those diameters were less than 3cm, the accurate localization of puncture under CT guidance may be more pivotal. Which means that higher disgnostic yield could be achieved so long as punture needles could successfully enter the lension and got the biopsy specimen, no matter how large the size of the lesion was. A lower disgnostic yield was met by R-EBUS because the ultrasonic probe’s scope is steady and changeless, so relative larger lesions could be more easily detected.

Data in Table 3 also indicated that the longer the distance from the SPN to pleura was, the higher positive disgnostic rate could be obtained. Recent reserch by Steinfort DP et al proposed the distance from hilum to lesion was a significant predictor of EBUS visualization yield [16]. In accordance with the previous reports we found the distance from location of SPN to pleura was related to the diagnostic yield in EBUS group [18, 23]. The possible reason may be that the shorter the distance to pleura was, the SPN is nearer to the peripheral distal bronchus. When the biopsy forcep were sent forward under the guidence of R-EBUS, the tip of forcep often could hardly fully open the narrowed lumen, so that invalid sampling and poor diagnostic yield might be resulted in.

**Figure 2:**
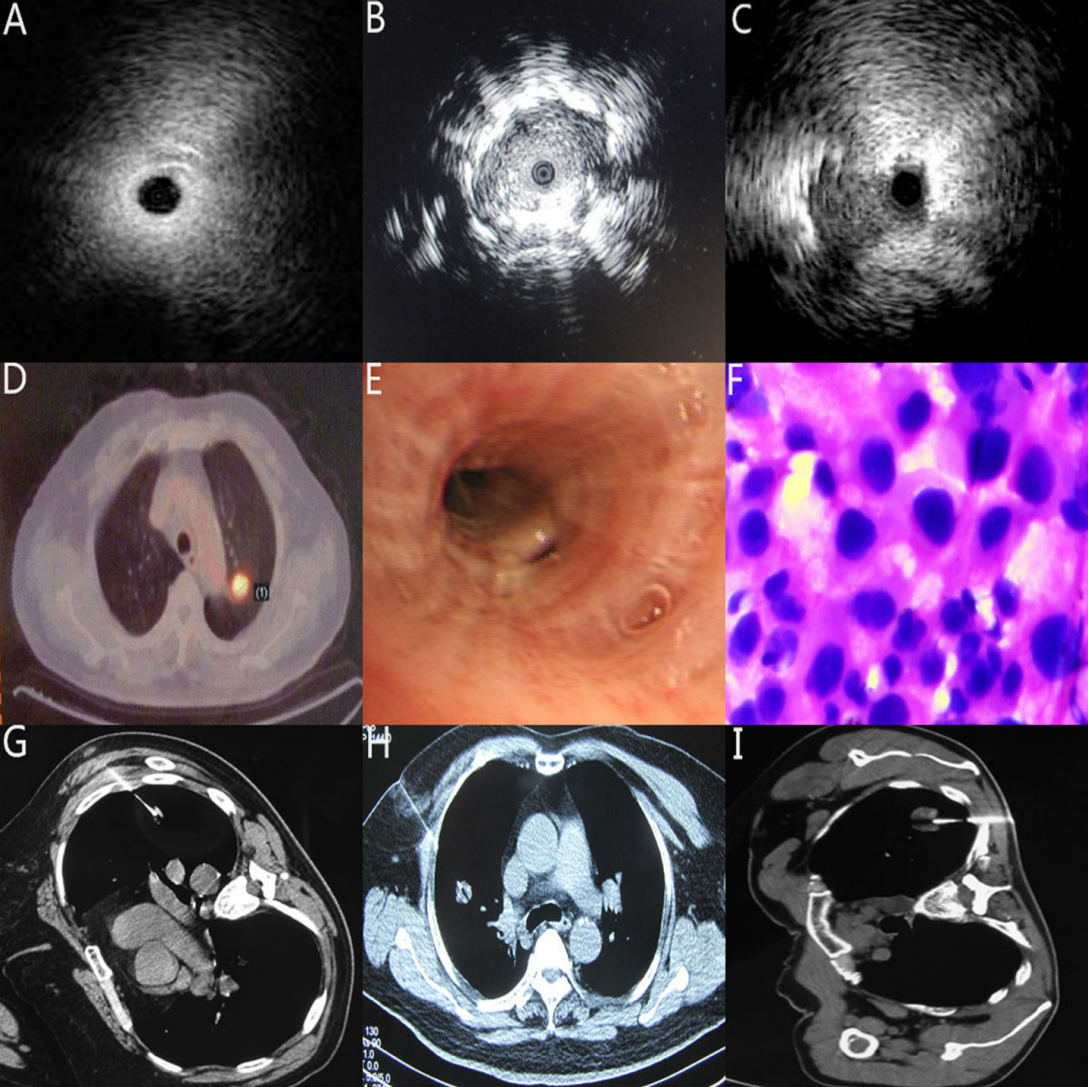
Typical image (**A**) The location of the probe was invisible to the SPN lesion (**B**) The location of the probe was within the SPN lesion (**C**) The location of the probe was adjacent to the SPN lesion (**D**) the SPN lesion located adjacent to aortic arch in PET-CT image photo with the approximate diameter ≈ 1mm. (**E**) the same patient as D, the SPN lesion was not seen in the view of conventional bronchoscopy. (**F**) the same patient as D, with the guidance of R-EBUS, the specimen from Bronchoscopic brushing was confirmed adenocarcinoma (**G**) Typical SPN with the diameter ≈ 1 mm in CT-PNB group (**H**) Typical SPN with the diameter ≈ 2 mm in CT-PNB group (I) Typical SPN with the diameter ≈ 3 mm in CT-PNB group.

Many studies have reported the position of probe could affect the diagnostic yield of R-EBUS [15, 16]. The data from our study also supported this point of view. We found The R-EBUS’ probe located within the SPNs had higher diagostic yield than those probes adjacent or invisible to the SPNs, which also supported the accepted viewpoint [14–16, 21]. As photos A-C showed in Figure 2, when the probe were positioned within the SPN lesion, the forcep could subsequently advance in right direction to obtain abundant specimen. When the probe only adjacent to or even invisible of the SPN image, the possiblity of invalid sampling will increase and negative diagnosis will be more likely to occur [22]. These data and statistical analysis proved the SPNs’ size, the distance from SPN lesion to plurea were two influencing factors for diagnostic yield in R-EBUS gourp.

The overall incidence of complications as pneumothorax and hemorrhage, in R-EBUS group, were 1.25% (1/80) and 5% (4/80). In CT-PNB group, the rate of pneumothorax was 17.5% (14/80), of hemorrhage, was 7.5% (6/80). Pneumothorax is one of the most common complications in lung puncture [8]. The incidence of pneumothorax in CT-PNB group was markedly higher than that in R-EBUS group. A closed drainage of thoracic cavity was performed in one patients only while oxygen therapy was used to cure the rest 13 patients. Meanwhile in R-EBUS group only one patient had slight pneumothorax.

Data in our study showed that in CT-PNB group, the longer the distance from the SPN to the pleura was, the higher the incidence of pneumothorax could be. This phenomenon could be explained by the fact the longer the distance from lesion to pleura was, the more pulmonary alveolus were possibly damaged by the needles passing through [8, 14, 24].

Our data showed the incidence of another common complication, pulmonary hemorrhage between the R-EBUS and CT-PNB group were similar. Subgroup analysis listed in CT-PNB group demonstrated that different distances from the SPNs to the pleura had different incidences of pulmonary hemorrhage. However, no similar changes were found in R-EBUS group. This phenomenon may be explained that the longer the distance from the SPN to pleura was, the more pulmonary tissue and vessel may be hurted by puncture needle. The R-EBUS was performed in human natural bronchus lumen so that such injury could be avioded. Among 6 cases with pulmonary haemorrhage in CT-PNB group, 5 cases were found their SPN lesions were near the great vessels. It indicated the richer the blood supply to SPN was, the more easily the pulmonary hemorrhage took place, which was also supported by some recent literature [8]. Our further analysis also indicated in CT-PNB group, both the distance from the SPN lesion to plurea, location of SPN was near to large blood vessels, were two influencing risk factors for pneumothorax and pulmonary haemorrhage.

In conclusion, both R-EBUS and CT-PNB are valuable tools for diagnosis of SPNs. Those SPNs location within the branches, near to the hilum or great blood vessels in chest, may be suittable for R-EBUS. Those SPNs located in peripheral lung field, near to pleura or lack of great vessels around may be fit for CT-PNB. It is important for clinicians to select strictly appropriate patients with SPNs for achieving high diagnostic yield and low incidence of complications.

There are several limitations in our study. First, the study was performed by a single medical institution. Second, different skills and proficiency levels of the three endoscopists participated in this study could produce bias. Third, the size of SPNs and the distance between the lesion and pleura were measured in line, which possibly cause bias compared with the measurement in three-dimensional forms. Strictly controlled, randomized and multi-center clinical research trails are still needed in the future [10].

## MATERIALS AND METHODS

### Description of randomization process

We have adhered to Optimization scheme of randomized trials of non-pharmacologic Treatment as the CONSORT guidelines showed [10, 25, 26].

### Calculation for sample size

Sample size in our study was estimated by algorithm via Inequality Tests for Two Proportions in the menu of PASS 8.0 software. with power 1-β = 0.90, Inspection level α = 0.05. According to the preset parameters, the PASS 8.0 software calculates that the two groups need 80 cases respectively. Therefore, this study expects two groups to have 80 cases respectively, which can ensure the accuracy and scientific results of the study.

The actual procedure are as follows: a computer engineer first generated a random number table (see in response letter, attachment 1) with the SAS 9.4 software, then another information engineer gave 1–160 Figures to each patient in the order of time when they were admitted to hospital. The recruited patients were divided into two groups (A:R-EBUS group and B:CT-PNB group) according to the random number table.

### Site of trial

Bronchoscopies and percutaneous lung puncture were conducted at the Endoscopic Center of Nanjing Chest Hospital.

### Manipulators

The examination was performed by three experienced endoscopists in turn.

### Patients

215 patients with SPN detected by spiral CT were consecutively enrolled from June 2014 to June 2016. According to the inclusion/exclusion criteria, finally 160 eligible patients were randomly allocated into either R-EBUS or CT-PNB group by random number selection. Before the operation all patients received lung and heart function, electrocardiogram and coagulation testing. Assessment about characteristics of SPN images in high resolution CT were recorded by two experienced radiologists. After the operation, all patients received a 24-hour medical observation for handle complications if necessary. Patients who were non-diagostic by R-EBUS or CT-PNB received VATS operation or antituberculous or antifungal therapy. All patients were followed up for at least 1 year [8]. The location on CT scans was defined as Central SPNs and Peripheral SPNs as described as the previous literature [19].

### Inclusion criteria

The characteristic of lung lesions accorded with the definition of SPN. Clinical and imaging data were visible. Patients who agree to sign informed consent.

### Exclusion criteria

Severe emphysema, multiple or single bullae in lung parenchyma near to SPNs. cardio or pulmonary function insufficiency. hemorrhagic diseases or coagulation disorders. the diameter of SPN < 8 mm whether anywhere in lung fields. The patient underwent mental disorders or those can not cooperate the examination.

### The procedure of Radial endobroncheal ultrasonography guided bronchoscopy

All bronchial were performed via nose route under local anesthesia and moderate sedation. An video bronchoscope (BF-P160, Olympus, Tokyo, Japan) equiped with a 20-MHz radial EBUS probe (UM-BS20e26R; Olympus, Tokyo, Japan) advanced along bronchus then the radial probe were inserted forward until the ultrasonic image indicating the target lesion clear. The radial ultrasound probe position were divided into three patterns as previously reported [14–16, 21]. (a) invisible: abnormal echogenicity was not completely detected; (b) within: entire circumference of abnormal echogenicity was detected; (c) adjacent to: partial circumference of abnormal echogenicity was detected. Then the probe was removed out and the deepth of ultrasound was marked to giude biopsy, brush and bronchoalveolar lavage sequentially. The histology, cytology and lavage fluid specimans were sent for patological examination timely.

### The procedure of computed tomography-guided percutaneous needle biopsy

Patients lay on inpection table in supine, prone, side or other position for finding out the shortest distance from the SPN lesion to the body chest surface. CT images were controlled to scan by a slice thickness of 5 mm to determine the puncture point, depth and direction. After marking on body surface, sterilization and local anesthesia, an introducer needle (Lot Number, REXK0682; Bard Peripheral Vascular, Inc., Tempe, AZ, 15 or 7 mm in length) was inserted into the SPN lesion under CT guide. If CT image comfirmed the tip of the puncture needle had entered the lesion, the patient was advised to hold his/her breath, then a cutting needle was stretched into the lesion via the introducter trocar. Repeatedly sampling was performed till appropriate specimens had been obtained. The procedure may be stopped by obvious cough, chest pain or any other discomfort symptom. After needle removal, CT scanning were carried out to detect if any complication such as pneumothorax and haemorrhage occur, for necessary intervene.

### Diagnostic criteria

#### Positive standard

(a) Pathological results of specimen were determined malignancy or identified pathogens as tubercle bacillus, fungal spore or mycelium etcetera.

(b) Histopathological showed granulomatous/inflammatory and the size of SPN remarkably reduced or disappear in CT image after experimental standardized antituberculosis/antibiotic therapy.

### Negative results

(a) Histopathological considered inflammatory but no shrink in size of leison after sufficient antibiotic therapy and finally were proved to be malignant by surgical resection.

(b) Histopathological findings showed abnormal shape cell or suspicious malignant cell but later determined to be a benign lesion by surgical resection.

### Statistical analysis

Data analyses were performed using SPSS statistical software (SPSS version 17.0, SPSS; Chicago, IL, USA). Continuous variables were analysed using Student’s *t*-test, and dichotomous variables were analysed with the chi-squared or the difference in frequency or Fisher’s exact test when necessary. Logistic analysis were used to calculated relevance. A two-tailed *P*-value of less than 0.05 was considered significant.

## Abbreviations

SPN: Solitary pulmonary nodule
CT-PNB: CT guided percutaneous needle biopsy
R-EBUS: Radial endobroncheal ultrasonography guided bronchoscopy
VATS: Video assisted thorascopic surgery
